# Achalasia-induced reversible sinus node dysfunction in DSP/TNNI3 cardiomyopathy: an extracardiac cause of sinus pauses mimicking progression of genetic cardiomyopathy

**DOI:** 10.1093/eschf/xvag109

**Published:** 2026-06-30

**Authors:** Luigi Cutore, Giuseppe Valadà, Giuseppe Leonardi, Paolo Zappulla, Davide Capodanno

**Affiliations:** Division of Cardiology, Azienda Ospedaliero-Universitaria Policlinico ‘G. Rodolico—San Marco’, University of Catania, Via Santa Sofia, 78, Catania 95123, Italy; Division of Cardiology, Azienda Ospedaliero-Universitaria Policlinico ‘G. Rodolico—San Marco’, University of Catania, Via Santa Sofia, 78, Catania 95123, Italy; Division of Cardiology, Azienda Ospedaliero-Universitaria Policlinico ‘G. Rodolico—San Marco’, University of Catania, Via Santa Sofia, 78, Catania 95123, Italy; Division of Cardiology, Azienda Ospedaliero-Universitaria Policlinico ‘G. Rodolico—San Marco’, University of Catania, Via Santa Sofia, 78, Catania 95123, Italy; Division of Cardiology, Azienda Ospedaliero-Universitaria Policlinico ‘G. Rodolico—San Marco’, University of Catania, Via Santa Sofia, 78, Catania 95123, Italy

**Keywords:** Genetic cardiomyopathy, Arrhythmia, Atrial compression, Achalasia, Reversible sinus pauses

## Introduction

Genetic cardiomyopathies, including variants in TNNI3 (troponin I type 3), DSP (desmoplakin), and MMAB (methylmalonic aciduria type B), predispose to ventricular dysfunction, arrhythmias, and sudden cardiac death.^1–3^ Arrhythmias and syncope may occasionally result from extracardiac causes, including mechanical cardiac compression, which can mimic cardiomyopathy progression and be reversible.^1,2,4^

Oesophageal achalasia, a rare motility disorder characterized by impaired lower oesophageal sphincter relaxation, typically presents with dysphagia and chest pain, but may rarely cause cardiac manifestations via atrial compression.^5,6^ We report a young man with a genetic predisposition to dilated cardiomyopathy who developed syncope and sinus pauses secondary to achalasia, successfully treated with peroral endoscopic myotomy.

## Case presentation

A 31-year-old man with a family history of dystrophinopathy and dilated cardiomyopathy, on his father side, presented to our outpatient cardiology clinic for annual follow-up. He was a smoker, exercised regularly, reported anxiety-stress and was of normal weight. Never required hospital admission. Since the age of 26, he had experienced frequent episodes of palpitations, though without exertional dyspnoea (New York Heart Association class I).

Routine blood tests were within normal limits except for a mildly elevated creatine phosphokinase (CPK, 283 U/L). Transthoracic echocardiography revealed a left ventricle with mildly reduced systolic function (ejection fraction 50%, end-diastolic diameter 54 mm, end-systolic diameter 41 mm), and subtle wall motion abnormalities. Cardiac magnetic resonance confirmed mild left ventricular dilatation with ejection fraction 52%, hypokinesis of the mid-basal interventricular septum and non-ischaemic late gadolinium enhancement of the inferolateral wall. Genetic testing identified heterozygous variants in MMAB (c.398 C>T), TNNI3 (c.204 G>T), and DSP (c.88 G>A); genes known to predispose to dilated and arrhythmogenic cardiomyopathies.

Electrocardiogram showed sinus rhythm at 60 b.p.m. with frequent premature ventricular extrasystoles. Given the risk suggested by the genetic background therapy was initiated with bisoprolol, ACE inhibitor and antiplatelet.

The following year, because of recurrent palpitations, a 24-h Holter electrocardiogram was performed and revealed a high burden of ectopic beats (7594 premature ventricular contractions and 2287 premature atrial contractions). Considering the risk of arrhythmic progression amiodarone (200 mg daily) was added to the therapy and a loop recorder was implanted, documenting persistent ventricular ectopy despite antiarrhythmic therapy. The patient subsequently underwent an electrophysiological study that identified a premature ventricular focus located on the inferobasal interventricular septum, which was successfully ablated by radiofrequency energy (maximum power 50 W) performed from a retrograde arterial approach. No further ventricular ectopy was observed on loop recorder follow-up.

**Figure 1:**
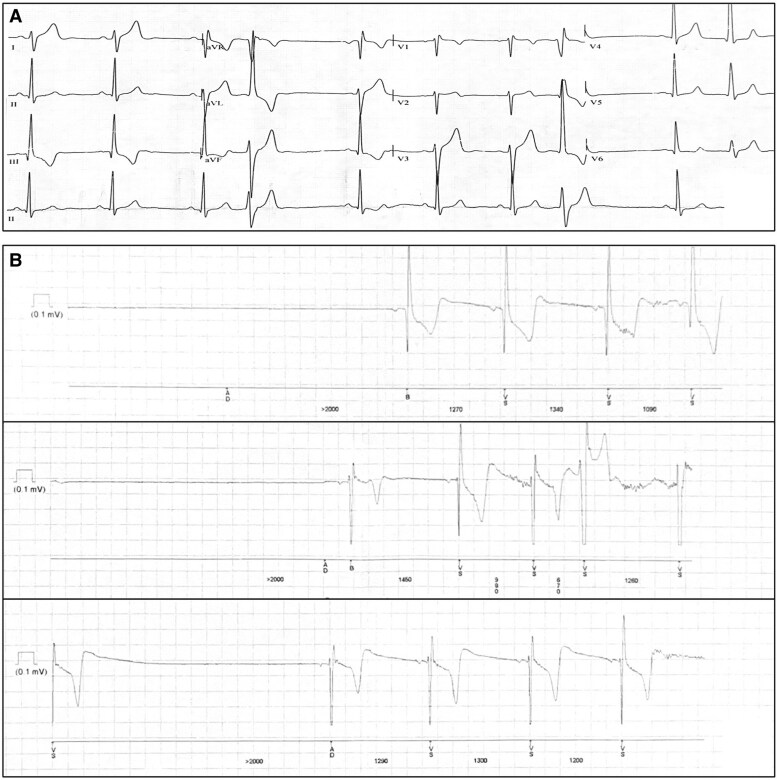
Electrocardiographic findings. (*A*) Baseline ECG showing burden of premature ventricular contractions; (*B*) Loop recorder tracing showing a sinus pauses

After two years of apparent clinical stability, the loop recorder unexpectedly documented asymptomatic recurrent nocturnal sinus pauses, the longest of which lasted over 6 s (*Figure 1*). This raised concern for progressive conduction system involvement as part of the underlying genetic cardiomyopathy, given the arrhythmic risk associated with TNNI3 and DSP variants. At the same time, however, the patient began reporting increased anxiety, easy fatigability, loss of appetite, dyspnoea, and recurrent postprandial syncopal episodes. These pauses raised consideration for permanent pacemaker implantation.

**Figure 2:**
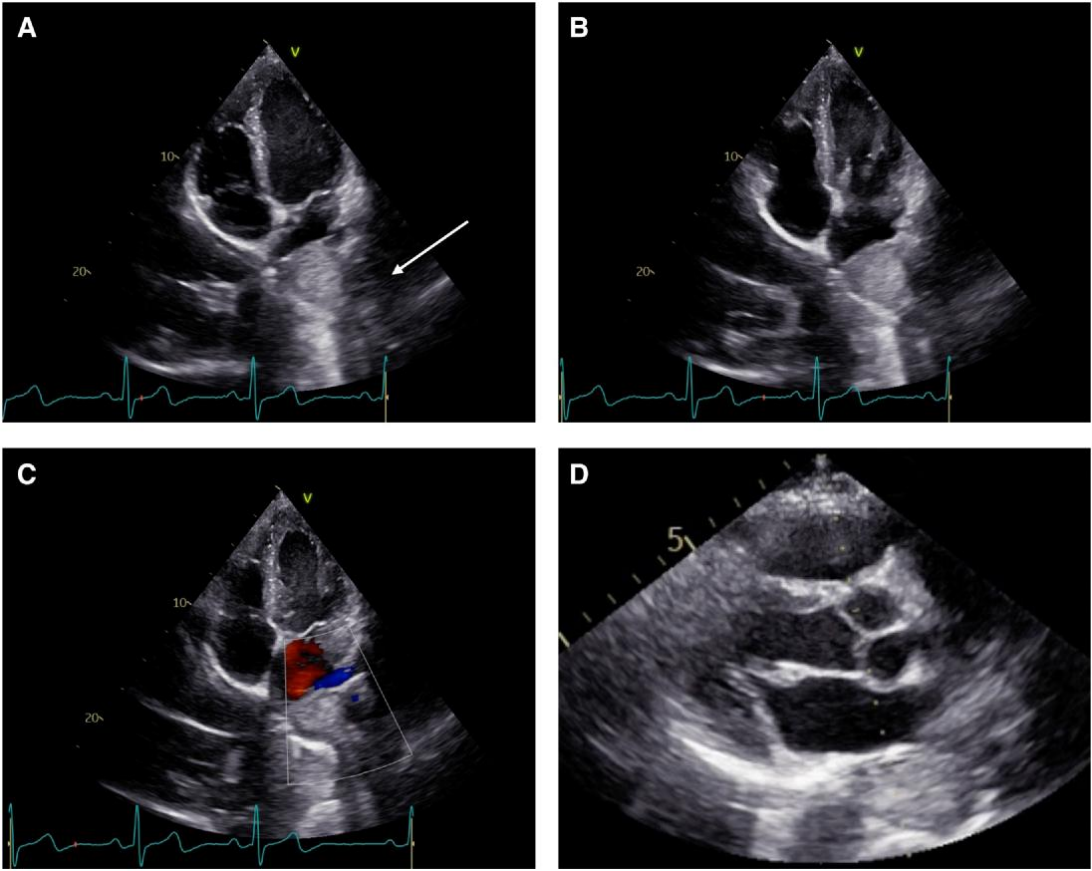
Echocardiographic findings. (*A*) transthoracic apical four-chamber view during tele-diastole showing an echogenic structure within the left atrium (arrow); (*B*) the same view during proto-diastole confirming that the structure compression persists; (*C*) colour Doppler imaging in the same projection showing no flow signal within the structure; (*D*) parasternal long-axis view demonstrating that the structure lies adjacent to, but not within, the left atrial cavity

**Figure 3:**
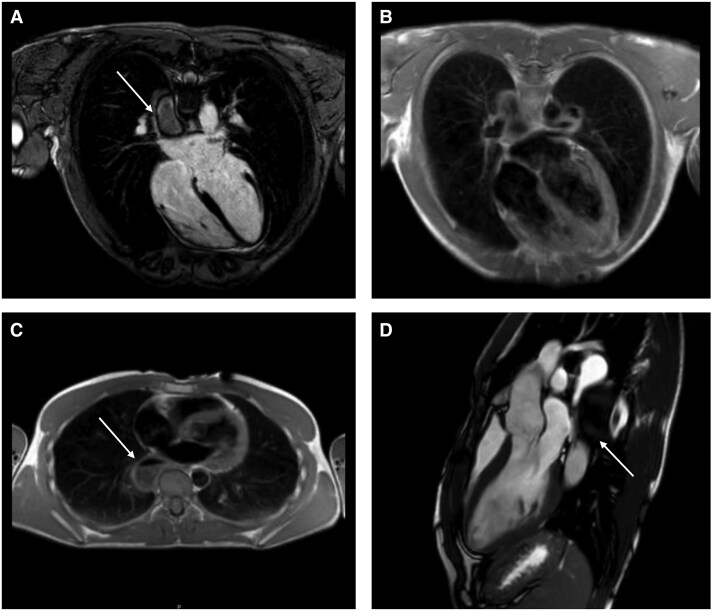
Magnetic resonance findings. (*A*) Cine steady-state free precession cardiac MR image (axial view) showing an apparent mass along the posterior wall of the left atrium (arrow). (*B*) T1-weighted black-blood axial image showing a structure (arrow) located posteriorly to the left atrium, with signal intensity consistent with the oesophageal lumen. (*C*) T1-weighted black-blood axial cardiac MR image demonstrating a tubular structure (arrow) posterior to the left atrium, consistent with the oesophageal lumen rather than an intracavitary mass. (*D*) Sagittal cine steady-state free precession image showing the left atrium and the adjacent bright oesophageal lumen posteriorly

Transthoracic echocardiography identified a hypoechoic, non-vascularized structure (35 × 24 mm) apparently compressing the left atrium, prompting further investigation (*Figure 2*). Repeat magnetic resonance showed progression of dilated cardiomyopathy with ejection fraction 46%, reduced global longitudinal and circumferential strain, and subendocardial late gadolinium enhancement of the basal inferior wall. No intracardiac mass was detected. Instead, imaging revealed a markedly dilated thoracic oesophagus extending to the gastroesophageal junction, compressing the posterior left atrial wall, the inferoposterior interatrial septum, the inferior vena cava and the right atrial junction (*Figure 3*).

This striking extracardiac finding shifted the diagnostic perspective. A barium swallow, esophagogastroduodenoscopy and oesophageal manometry confirmed the diagnosis of type II achalasia. Given the temporal association between meals, syncope, and documented sinus pauses, the working hypothesis was that mechanical compression of the atria by the dilated oesophagus was the main trigger of the arrhythmias.

The patient underwent peroral endoscopic myotomy. A temporary pacemaker was prophylactically implanted and removed after the intervention. Peroral endoscopic myotomy consisted of submucosal tunnelling followed by selective circular myotomy measuring approximately 8 cm in the distal oesophagus, with a 2 cm extension onto the gastric cardia, while preserving the longitudinal fibres to maintain structural integrity and oesophageal motility. The procedure was completed without complications and was well tolerated. After 3 days of fasting, control esophagogastroduodenoscopy confirmed adequate gastroesophageal junction patency and correct positioning of the mucosal clips, without leakage, allowing gradual refeeding with liquid and subsequently semi-liquid diet. Following the procedure, his symptoms completely resolved and the loop recorder documented disappearance of sinus pauses and arrhythmias. He continues regular cardiological follow-up for the underlying genetic cardiomyopathy.

## Discussion

This case illustrates the challenges in managing a young patient with genetic cardiomyopathy and arrhythmias, complicated by an extracardiac disorder. Heterozygous variants in MMAB, TNNI3, and DSP were consistent with a predisposition to dilated and arrhythmogenic cardiomyopathy, characterized by ventricular dysfunction and ventricular arrhythmias.^1,2,4^

The onset of nocturnal sinus pauses and postprandial syncope, initially attributed to progression of the cardiomyopathy, was found to be secondary to atrial compression by a dilated oesophagus due to achalasia.^5,7^ The nocturnal predominance of sinus pauses in this patient can be explained by the interaction between mechanical and autonomic mechanisms. Severe oesophageal dilatation has been reported to cause extrinsic cardiac compression and rhythm disturbances, providing a mechanical substrate for intermittent sinus node dysfunction. Superimposed on this, the physiological increase in vagal tone during sleep, known to reduce sinoatrial automaticity, may further lower the threshold for bradyarrhythmias, thereby enhance the impact of extrinsic compression on the sinus node.^8^

This case underscores the importance of considering extracardiac causes in patients with unexplained arrhythmias, even in the presence of a known genetic cardiomyopathy. Multimodal imaging allowed differentiation between cardiomyopathy progression and mechanical atrial compression, guiding targeted therapeutic decisions.

Desmoplakin and TNNI3 variants can lead to variable phenotypes, from isolated ventricular arrhythmias to overt dilated cardiomyopathy with risk of sudden cardiac death.^2,4,9^ Notably, pathogenic DSP variants have also been associated with sinus node disease and progressive conduction abnormalities, which initially supported consideration for permanent pacemaker implantation.^3^ However the coexistence with achalasia, incidental but clinically significant, was ultimately revealed to be decisive.^6^ Treatment with peroral endoscopic myotomy completely resolved symptoms and sinus pauses, allowing the avoidance of pacemaker implantation.^6^ This represents an example of reversible sinus node dysfunction, a phenomenon rarely reported, highlighting the importance of distinguishing functional from structural causes in patients with genetic cardiomyopathy.

In conclusion a comprehensive diagnostic approach is essential when patients with genetic cardiomyopathy develop new or atypical arrhythmic symptoms. In this patient, electrophysiological study and targeted ablation proved effective in suppressing ventricular ectopy, emphasizing the value of accurate arrhythmic substrate identification. It also demonstrates that extracardiac factors, such as atrial compression from a dilated oesophagus, can mimic progression of cardiomyopathy and lead to potentially reversible arrhythmias.

Recognition of these uncommon but clinically significant mechanisms can prevent unnecessary invasive interventions, such as pacemaker implantation and guide targeted therapy. Multimodal imaging and functional testing are essential to distinguish primary cardiac progression from secondary mechanical causes.

## Declarations

### Data Availability

No data were generated or analysed for this manuscript.
